# Gastric Adenocarcinoma in the Setting of IPEX Syndrome

**DOI:** 10.1155/2021/9967198

**Published:** 2021-06-25

**Authors:** David Steffin, Saleh Bhar, Douglas S. Fishman, Nicholas L. Rider, Bindi Naik-Mathuria, Caridad Martinez, Rajkumar Venkatramani

**Affiliations:** Texas Children's Hospital, Baylor College of Medicine, Houston, TX 77030, USA

## Abstract

Immune dysregulation, polyendocrinopathy, enteropathy, X-linked (IPEX) syndrome is a rare X-linked disorder caused by a loss of function mutation in the *FOXP3* gene. It manifests early in infancy with clinical symptoms including autoimmune enteropathy, type 1 diabetes mellitus, and eczema. While aberrant *FOXP3* expression has been associated with several types of cancer, little is known regarding the risk of cancer in patients with IPEX harboring the characteristic *FOXP3* mutation. Here, we present a unique case of a primary signet ring gastric adenocarcinoma in a pediatric patient with IPEX syndrome.

## 1. Introduction

Immune dysregulation, polyendocrinopathy, enteropathy, X-linked (IPEX) syndrome is a rare *X*-linked autoimmune disorder caused by a loss of function mutation in the *FOXP3* gene. Historically, patients did not survive past the third decade of life; however, improved supportive care and hematopoietic stem cell transplant has led to an improved survival nearing 73% [1].

Aberrant somatic *FOXP3* expression has been associated with various malignancies including breast, prostate, colon, and ovary [2]. Due to the rarity of IPEX and low life expectancy, the incidence of associated malignancy is unknown. Only one prior case report of a child with IPEX and hematologic malignancy has been described, a patient who developed EBV virus-induced lymphoma after treatment with rapamycin for IPEX-related symptoms [3]. Here, we add to that literature, presenting a child with IPEX syndrome with an identified mutation on the *FOXP3* gene that developed signet ring cell gastric adenocarcinoma.

## 2. Case Presentation

Our patient, a white Hispanic male, was initially born full term without any perinatal complications. He was discharged to home and soon after admitted to the intensive care unit for neonatal sepsis, and he was subsequently diagnosed with hyper IgE syndrome based on elevated IgE titer. He did well until 4 years of age, when he developed chronic gastritis with gastric ulcers. The patient was started on prednisolone and continued steroid therapy for 5 years, when he was switched to azathioprine as a steroid sparing agent. At 12 years old, his newborn brother was diagnosed with IPEX syndrome after developing diabetes, dermatitis, and chronic diarrhea. This prompted sequencing of the *FOXP3* gene in our patient, which revealed a hemizygous missense mutation resulting in a substitution of aspartic acid for asparagine missense mutation at amino acid 383 at the c.1147 locus (1147 G > A). This was the same mutation as his brother's, which confirmed the diagnosis of IPEX syndrome. Our patient's flow cytometric assay was reported normal. Results indicated a regulatory T-cell population (% of CD4) of 9.9% (normal range 4.2–9.9%), *FOXP3* percentage of 75% (normal range 75–81%), and an absolute *FOXP3* of 94 cells/mcL (normal range of 17–200).

**Figure 1:**
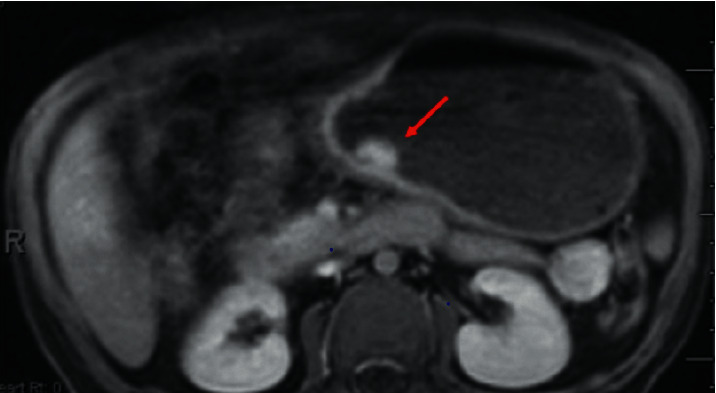
MRI of the abdomen and pelvis with and without contrast of patient demonstrating evidence, a 0.6 × 1 cm focal polypoid-like mass lesion was observed near the junction of the distal body/antrum of the stomach.

**Figure 2:**
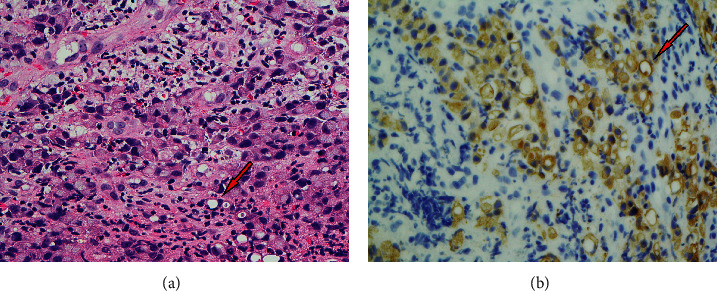
(a) H&E staining at 60x of signet cell gastric adenocarcinoma specimen obtained following total gastrectomy. Visualized are sheets of signet ring cells with eccentrically located hyperchromatic nuclei in the lamina propria in a background of acute gastritis with reactive epithelial changes of the surface epithelium. (b) Pancytokeratin staining at 60x after immunohistochemistry of the patient's biopsy specimen, which is consistent with the patient's diagnosis of signet ring cell adenocarcinoma.

At 14 years of age, the patient presented with a one-month history of progressive intolerance to solid foods resulting in vomiting and weight loss. *H. pylori* testing was negative. MRI of the abdomen revealed a 1.6 × 1 cm focal polypoid-like mass lesion near the junction of the distal body/antrum of the stomach along the greater curvature with no extramural extension (Figure 1). Endoscopic biopsy of the mass demonstrated pathology consistent with signet ring cell adenocarcinoma (Figure 2). Ultrasound, staging laparoscopy, and perigastric lymph node biopsy were performed, and they revealed a stage II gastric cancer. He received 6 cycles of neoadjuvant 5-fluorouracil and oxaliplatin chemotherapy (FOLFOX regimen). He tolerated chemotherapy without serious infectious complications.

Five months after diagnosis, he underwent total gastrectomy with Roux-en-Y esophagojejunostomy including D2 lymphadenectomy and jejunostomy-tube placement. Three of the 21 lymph nodes sampled were positive for tumor. The overall stage was IIA according to the American Joint Committee on Cancer Staging. His postoperative course was uncomplicated except for exacerbation of colitis and inability to tolerate enteral feeds. There were no infectious complications. He received two cycles of FOLFOX postsurgery. Due to concern for exacerbation of colitis, he received maintenance oral capecitabine for six months in lieu of consolidative abdominal irradiation. He is in remission 23 months from diagnosis.

## 3. Discussion

Malignancies are rare in patients with IPEX syndrome. Only one prior report of a child with IPEX and malignancy has been described: an EBV virus-induced lymphoma after treatment with rapamycin. This patient developed cervical lymphadenopathy after 8 months of treatment with rapamycin. Biopsy confirmed CD20+ diffuse large B-cell lymphoma. He was refractory to three rounds of anti-CD20 monoclonal antibody and went into remission following treatment with cyclophosphamide and vincristine [3].

Gastric cancers in pediatric patients in general are also extremely rare. In a report on 5 pediatric patients with gastric adenocarcinoma, all patients presented with nonspecific symptoms with the most common being vomiting, abdominal pain, anemia, and weight loss. Four of the five patients had diffuse metastatic disease. All patients received a platinum-based therapy with either cisplatin or oxaliplatin as well as 5-fluorouracil. Of the five patients, only one was reported alive at the time of publication. The mean time to death of the other patients was 2.8 months [4].

IPEX syndrome results from a pathological variant in the *FOXP3* gene leading to malfunction or absence of T-regulatory cells (Tregs). Interestingly, as in the case of our patient, there are circumstances in which patients might present with symptoms of IPEX syndrome with normal flow cytometric results. In a series of 28 patients evaluated for IPEX and IPEX like-syndromes, the flow cytometric results did not demonstrate abnormally low *FOXP3*-expressing cells in the peripheral blood. When evaluating the mean fluorescence intensity, there was weaker *FOXP3* expression, perhaps secondary to the location of the *FOXP3* mutation [5, 6].

*FOXP3*'s expression is not exclusive to Tregs as it is also detected in effector T-cells. Others have shown that *FOXP3* dysfunction in IPEX patients, regardless of the site of mutation, can lead to impaired cytokine production and effector T-cell function [6, 7].

Aside from the presence of Tregs, multiple immune infiltrates have been correlated with tumor progression in gastric cancers. In fact, high quantities of tumor-associated macrophages (TAMs), otherwise known as M2-type macrophages, have been associated with solid tumor progression and are thought to inhibit cytotoxic T-cell function via the production of anti-inflammatory cytokines (i.e., IL-4, IL-10, and IL13), as well as the promotion of tumor angiogenesis [8–10]. Additionally, TAMs can enhance the expansion of myeloid derived suppressor cells (MDSCs) by the production of granulocyte/macrophage colony-stimulating factor, vascular endothelial growth factor, and interleukin-6. Once MDSCs expand and infiltrate gastric tumors, they are activated via interleukin-4 and 13 and transforming growth factor-*β*, among other inhibitory cytokines, thereby releasing various reactive oxygen species, arginase-1, and inducible nitric oxide synthase to inhibit cytotoxic T-cell function [11]. While we were unable to test for immune infiltrates in our patient's biopsy sample, both TAM and MDSC infiltration are already known to be correlated with more aggressive gastric cancers. Though the literature is scant on their relative contributions to Treg infiltrates in tumor progression, we postulate that their involvement, along with likely impaired effector T-cell function due to the patient's known *FOXP3* mutation, played an active role in the progression of this patient's signet ring adenocarcinoma.

In addition to *FOXP3*'s role in immune cell function, genetic analysis of *FOXP3* mutations suggests that locations commonly associated with more aggressive phenotypes of IPEX syndrome share similarity in the location of somatically acquired mutations of *FOXP3*-related cancers [2]. This suggests that *FOXP3* may act as an X-linked tumor suppressor, and thus, its under expression might promote tumor survival [12]. While direct links between IPEX and tumor malignancy remain unclear, the *FOXP3* gene has already been studied in the context of malignancy, with some links between somatic mutations of *FOXP3* in epithelial cells and increased susceptibility to breast cancer [13]. One mechanism is that impaired *FOXP3* leads to enhanced NF kappa beta signaling which promotes angiogenesis, metastasis, and poor differentiation of cancers [14].

We hypothesize that this patient's IPEX syndrome led to the development of his signet ring adenocarcinoma, via the disruption of *FOXP3* both through its function as a potential tumor suppressor gene and its concomitant role in both regulator and effector T-cell homeostasis. As there are currently no recommendations for surveillance of malignancy in patients with IPEX syndromes, further research is needed to both identify the risk of cancer and further elucidate the tumor microenvironment in this patient population.

## Data Availability

The data submitted for this case report include basic vital signs, blood tests (blood counts, chemistries, and genetic testing), and imaging results. These data have been deidentified and are restricted given that they contain confidential patient information. All relevant testing is included within the body of the text. The data used to support the findings of this study are available from the corresponding author upon request.
